# Suicidal behavior and associated factors among prisoners at Dessie town correctional institution, Dessie, Ethiopia

**DOI:** 10.1186/s12888-022-04306-2

**Published:** 2022-10-24

**Authors:** Tamrat Anbesaw, Million Tsegaw, Abubekr Endra

**Affiliations:** Department of Psychiatry, College of Medicine and Health Science, Wollo University, P.O. Box 1145, Dessie, Ethiopia

**Keywords:** Suicidal behavior, Depression, Prison, Dessie, Ethiopia

## Abstract

**Background:**

Suicide is a prominent cause of death among inmates worldwide, accounting for over 30% of all deaths. Several factors, including prison-related, psychiatric disorders, stressful life events, and substance use-related factors are believed to be associated with an increased risk of suicidal behavior in a correctional facility. The present study aimed to determine the prevalence and associated factors of Suicidal Behavior among prisoners at Dessie town correctional institution, Ethiopia.

**Methods:**

From February 16 to March 5, 2020, a cross-sectional survey was conducted among 288 inmates at the Dessie Town Correctional Center. A systematic random sampling technique was used to select study participants during the study period. Data was collected through face-to-face interview methods using Suicidal Behavior Revised (SBQ-R). The collected data were coded, entered into Epi-data data version 3.1, and analyzed by SPSS Version 26. Binary logistic regression was carried out to identify independent predictors of suicidal behavior at a 95% confidence level. Variables at a *p*-value < 0.05 with 95% CI were declared statistically significant.

**Results:**

The prevalence of Suicidal behavior among prisoners was found to be 25.3% [(95% CI: 20.5, 30.6)]. This study showed that being female [AOR = 5.14;95% CI (1.62,16.29)], depression [AOR = 4.97;95%CI (2.53,9.77)], anxiety [AOR = 3.14; 95%CI (1.59,6.22)], experienced stressful life events [AOR = 5.11; 95%CI (2.24, 11.65)], and ever substance use [AOR = 2.83; 95%CI (1.41, 5.59)] were variables significantly associated with suicidal behavior among prisoners in Dessie town correctional institution.

**Conclusion and recommendations:**

In this study, suicidal behavior was highly prevalent among prisoners compared to the general population. Being female, depression, anxiety, stress full life events, and substance use were variables that are independent predictors of suicidal behavior. This study recommends that the institution needs to deliver an appropriate psychiatric facility to diagnose and treat prisoners with suicidal behavior. Also, special attention should be given to early screening and treatment of prisoners through prison health services, which is the most critical prevention strategy of suicide in prisoners.

## Background

Suicidal behavior is a fatal act that represents the person’s desire to die [1]. There is a range between thinking about suicide and acting it out. Some plan for days, weeks, or even years before acting, while others take their lives seemingly on impulse without premeditation. It is a complicated process that includes suicidal ideation, suicide planning, suicide attempt, and suicide completion [2]. According to the World Health Organization (WHO) report, over 16 million individuals attempt suicide each year, and over 1 million people die by suicide every year, with low and middle-income countries (LMICs) accounting for 75% of those who die by suicide. Suicide ranked as the 14th leading cause of death and morbidity in the world. It is anticipated to rise by 50% by 2030, making it the 12^th^ biggest cause of mortality [3], and one of the main causes of death among prisoners [4].

The prison is a correctional facility where convicts' liberty, autonomy, and communication with family and friends are restricted. This can be overwhelming to some prisoners, thereby leading to a deterioration in their physical, psychological, and social well-being [5]. Worldwide, more than 10.1 million people are currently incarcerated in correctional institutions. Around 50% of the world's prisoners are from the United States (2.29 million), China (1.65 million sentenced inmates), and Russia (0.81 million), and most of them are incarcerated from minority groups or low and middle-income countries (LMIC) [6].

Suicidal behavior is one of the most common causes of death in the United States, accounting for 35,000 deaths each year. It is also the third-largest cause of death in US prisons [7]. This makes suicide the leading cause of death among inmates and it is a public health concern [8], occurring at a 3–8 times higher rate in prison than in the general community [9]. The national cost of suicide in the United States in 2013 was 58.4 billion dollars [10, 11]. On the other hand, in England and Wales, male and female prisoners are a particularly vulnerable group, with 6 times and 20 times excess of suicides in prisoners compared with the general population respectively [12].

Research conducted in the USA describes the lifetime prevalence of suicide ideation among prisoners as 19.0%, and suicide attempts were reported by 11.9% [13]. In addition, in New York state correctional institutions, 34% of participants had expressed suicidal ideation and 64% of them had attempted suicide [9]. Another cross-sectional study found that 33.7% of prisoners in New South Wales, Australia, had suicidal ideation, and 20.5% had attempted suicide [14]. Studies showed in Africa, Nigeria, the weighted prevalence of suicidal ideation was 7.28% [15]. In studies from Ethiopia, a month prevalence of suicidality in Addis Ababa correctional center was 8.04% [16]. Another study from Jimma prison, Ethiopia, showed the overall prevalence rate of suicidal behavior was 23.2% [17]. In a study conducted in North West Amhara Regional State, Ethiopia, the prevalence of suicidal ideation, and planned to commit suicide were 17% and 16.6% respectively [18].

In an attempt to explain the links between substance abuse, depression, anxiety, and suicidal behavior, a number of hypotheses and theories have been proposed. Substance use (e.g. acute intoxication that increases impulsivity or disinhibition and affects judgment and problem-solving abilities) is thought to be a vulnerability or propensity (diathesis) to suicidal behavior, according to biological theories. Stressful circumstances (such as a depressive episode or a relationship disagreement) operate as triggers in this concept, leading to suicidal behavior. Problematic substance use can have negative effects on social integration and disrupt social regulation, leading to suicidal behavior [19]. Different factors such as being female, poor social support, family history of mental illness, unmarried, repeated incarceration, young age, low educational status, perceived stigma, poor of social support, duration of stay, non-employment, comorbid mental disorders, usually depression and substance use may increase the probability of having suicidal behavior [16, 19–24].

Suicidal behaviors are common in correctional institutions, and less attention has been paid to this public health problem, especially in middle‑ and low‑income countries including Ethiopia. Even though the national health policy of Ethiopia gives attention to health care delivery at the community level, still it is not possible to address those who are suffering from suicidal behavior particularly in prisons; its impact and prevalence are still underestimated and underreported among prisoners in our country. There were no sufficient studies conducted in Ethiopia, especially in northeast parts to determine the prevalence of suicidal behavior and its associated factors among those incarcerated in prison. Also, the previous studies conducted in Ethiopia, not assess factors such as anxiety, substance use, and stressful events among prisoners. Therefore, this study aimed to assess the prevalence and associated factors of suicidal behavior among prisoners in Dessie town correctional institution. This study will show the severity to policymakers and different stakeholders to integrate mental health services within the prisons to prevent suicide. Additionally, it will also serve as baseline data for future studies and researchers.

## Methods and materials

### Study setting

The study was conducted in Dessie City Correctional Center, Northeast, Ethiopia, from February 16 to March 5, 2020. Dessie town has one correctional institution, which was established in 1958. This correctional institution is located in South Wollo, Amhara Regional State, in North-Eastern Ethiopia, 401 km from Addis Ababa, Ethiopia's capital, and 482 km from Bahir Dar, Amhara Regional State's headquarters. The city contains 18 kebeles with a total population of 219,978 people (99,822 male and 120,156 female), according to data from the South Wollo Zone statistics office for 2016–17. According to global jail population data from 2015, Ethiopia's population is incarcerated to the tune of 111,050 people [25]. During the study period, there were about 1250 prisoners. There is only one clinic that serves inmates in prison, and it does not provide psychiatric services. Murder, physical assault, attempted murder, rape and abduction, theft and robbery, and political concerns all result in intimates being imprisoned.

### Study design

An institutional-based cross-sectional study design was conducted.

### Population

#### Source population

All adult prisoners (18 years and above old) in Dessie city correctional center.

### Study population

All adult prisoners at the Dessie Correctional Institution were included in the sample during the data collection period.

Prisoners who were critically ill and unable to communicate at the time of data collection.

### Eligibility criteria

In our study, the inclusion criteria were all prisoners age 18 and above while prisoners who were critically ill and unable to communicate during data collection were excluded from the study.

### Sampling procedure and sampling techniques

#### Sample size determination

To determine the sample size for the study population the following assumption was made. The actual sample size for the study was determined by using a single population proportion formula, by assuming a 5% margin of error and 95% confidence interval at alpha (α = 0.05), and the population proportion (23.2%) was taken from a study conducted at Jimma correctional institution [17]. So based on the above information the total sample size was calculated as:

So, the sample size was determined as;

$$\mathrm n=\frac{\left(\mathrm Z\;\mathrm\alpha\;/2\right)^2\mathrm P\left(1-\mathrm P\right)}{\mathrm d^2}=\quad\quad\quad\mathrm n=\frac{\left(1.96\right)^2\times\left(0.232\left(1-0.232\right)\right)}{\left(0.05\right)^2}=274$$

Where n = sample size;

ᾳ = confidence interval of 95% = 1.96.

p = prevalence of suicidal behaviour 23.2% (Study done in Jimma correctional institution).

d = marginal error = 0.05.

Therefore, the final sample size by adding 10% non-respondent = 302.

#### Sampling technique

After the sampling frame was developed, a systematic random selection process was employed to choose study participants. The sampling interval (K) was obtained by dividing the study population with the final sample size as follows; 302 $=\frac{N}{n} , k=\frac{1250}{302}=4.02=$ 4. As a result, the actual participant was drawn at random every four intervals in the sampling frame until the desired sample size was reached. The first study subject was chosen by lottery from a list of 1–4 candidates. As a result, starting with the first study unit, volunteers were chosen every four intervals.

### Operational definitions

#### Suicidal behavior

Incarcerated people who scored SBQ-R ≥ 7 were labeled as high-risk, and those below 7 as low-risk [26].

#### Depression

Was assessed using the hospital anxiety and depression scale (HADS). Participants who scored > 8 were considered to have depression [27].

#### Anxiety

Using the hospital anxiety and depression scale (HADS), normal (score < 7) and having anxiety (score > 7) [27].

#### Stressful life events

Defined as experiencing one or more stressful life events in the last 1 year [28, 29].

#### Social support

Using the Oslo-3 scale, prisoners with a mean score of < 8.1 (poor social support), > 8.1 (good support) [30].

#### Ever substance use

Use of at least one substance (Alcohol, tobacco, and khat use) in a lifetime [31].

### Data collection method and instruments

Data were collected using an interviewer-administered questionnaire, which has four subunits. Socio-demographic factors, clinical factors, psychosocial, and substance-related factors were developed after an extensive review of the literature and similar study tools.

Suicide behavior in prisoners was measured using the Suicidal Behavior Questionnaire-Revised, which included the four items listed below (SBQ-R). SBQ-R item 1 assess lifetime suicidal thoughts and attempt, item 2 assesses the frequency of suicidal ideation over the last year, item 3 defines the threat of suicide conduct, and item 4 assesses self-reported future suicidal behavior probability. Its sensitivity and specificity were 80% and 91%, respectively, on a scale of 3 to 18. The presence of suicidal behavior among prisoners in community settings is explained if a score ≥ 7 for SBQ-R [26]. In addition, from question number one, it was classified into having suicidal ideation, plan/intent, and attempt for discussion purposes. Suicidal ideation (If the respondent answers the question have you ever thought/ brief passing thought about committing suicide? If yes, the patient has suicidal ideation) [26, 32]. Suicidal plan/intent (If the respondent answers the question have you had a plan at least once to kill yourself? If yes, the patient has a suicidal plan /intent) [32]. Suicidal attempt (defined as; if the respondent answers the question have you ever attempted to kill yourself? If yes, the patient has a suicidal attempt) [32]. The internal consistency (Cronbach alpha) of (SBQ-R) in this study was 0.83.

The questionnaire contains variables used to assess dependent variables, including socio-demographic factors (age, gender, religion, ethnicity, marital status, educational status, occupational status, clinical factors (depression and anxiety), and psychosocial factors (social support, stressful life events, length of stay, and ever use of a psychoactive substance [31].

The hospital anxiety and depression scale (HADS) is made up of two subscales, one of which assesses depression and the other of which assesses anxiety. Respondents scored each item on a four-point (0–3) scale, resulting in possible scores ranging from 0 to 21 for each of the two subscales [27]. A score of 0 to 7 is considered "normal" according to the HADS manual. In a study comparable to others, a cutoff point of > 8 was applied for depression and anxiety measurements [33]. In this study, the internal consistency was 0.79 for depression subscales and 0.81 for anxiety.

The Stressful Life Events Screening Questionnaire (SLESQ) is a self-report assessment for exposure to one or more stressful life events. Events such as being unemployed, marital difficulties, spouse, child, or parent died, etc. For each event, respondents are asked to indicate whether the event occurred by ("yes" or "no") [28, 29]. In this study, the internal consistency (Cronbach alpha) of stressful life events was 0.81. The scale Oslo-3 was used to measure social support [30]. In this study, social support is measured based on the mean score of participants. Prisoners with a mean score of 8.1 or lower received poor social support, while those with a score of 8.1 or higher received good support. The internal consistency (Cronbach alpha) of Oslo-3 social support in this study was 0.87. The internal consistency (Cronbach alpha) of Oslo-3 social support and stressful life event in this study were 0.87 and 0.81 respectively.

### Data quality control

Data were collected through face-to-face interviews by trained three psychiatric nurses and one supervisor from the Integrated Clinical and Community Mental Health (ICCMH). The questionnaire was properly prepared and modified to ensure quality, and it was translated into the local language (Amharic) so that all participants could understand it and then translated back to English. The data collectors and supervisor have received training for two days duration on the purpose of the study, tools, how to collect data, sampling techniques, and how to handle ethical issues including confidentiality. A pre-tested on 5% in Kombolcha town correctional center one week before the main data collection to identify potential problems in the proposed study, such as data collection methods and data collector performance, and was not included in the main study. The data collectors were supervised regularly, and the supervisors and lead investigator examined the field questionnaires daily. The collected data were edited and entered into the computer from a paper then checked twice and processed timely.

### Data processing and analysis

Data were collected, cleaned, and stored for consistency on a computer using Epi-Data version 3.1, and then exported to SPSS 26 version statistical software for analysis. The researchers employed frequency, proportion, and other descriptive statistics. To identify independently associated factors for suicidal behavior, binary logistic regression was used to determine the association between explanatory factors and the outcome variable, and then all independent variables with a *p*-value less than 0.25 were entered in the final model (multivariable logistic regression). Hosmer Lemeshow's goodness fittest was used to assess the fitness assumption. AORs with 95% CI were used to measure the association's strength. Finally, variables with a *P*-value < 0.05 was considered statistically significant.

## Results

### Socio-demographic characteristics of the study participants

A total of 288 participants were involved in this study, making the overall response rate of 95.3%. The mean age (± SD) of the respondents was 30.11(± 7.89), with an age range of 18–51 years. Among the study participants, 266 (92.4%) were males and 132 (45.8%) were single. The majority of the participants 258 (89.6%) were Amhara by ethnicity and 184 (63.9%) were Muslim in their religion. From the study participants, 119(41.3%) were primary school and 90 (31.3%) of the participants were farmers by their occupation (Table 1).

**Table 1:** Socio-demographic characteristics of prisoners in Dessie city correctional institution, Dessie, Northeast, Ethiopia, 2020 (*N* = 288)

Variables	Category	Frequency	Percentage (%)
Age	18–27	122	42.4
	28–37	114	39.6
	38–47	43	14.9
	> 47	9	3.1
Sex	Male	266	92.4
	Female	22	7.6
Marital status	Married	106	36.8
	Single	132	45.8
	Divorced/separated	50	17.4
Ethnicity	Amhara	258	89.6
	Oromo	16	5.6
	Tigrie	14	4.8
Religion	Orthodox	84	29.2
	Muslim	184	63.9
	Protestant	16	5.6
	Others^a^	4	1.3
Educational status	Unable to read and write	38	13.2
	Primary	119	41.3
	Secondary	62	21.5
	College and above	69	24.0
Occupation	Farmer	90	31.3
	Governmental employed	31	10.5
	Non-government employer	30	10.4
	Merchant	57	19.8
	Student	47	16.5
	Other^b^	33	11.5

Others ^a^Juhabwitness

^b^Retired

### Clinical characteristics of the study participants

**Fig. 1:**
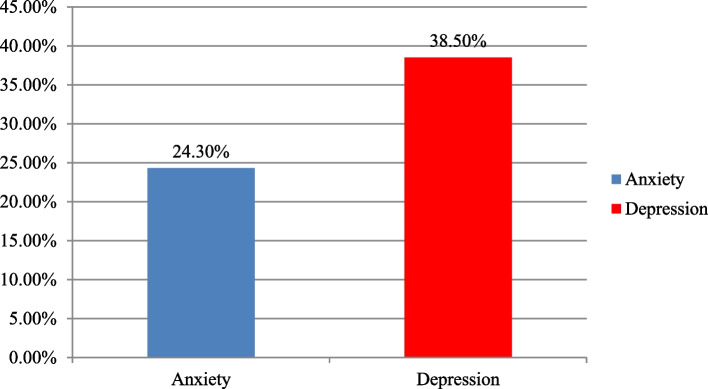
Clinical characteristics of prisoners in Dessie city correctional institution, Dessie, Northeast, Ethiopia, 2020 (*N* = 288)

Among the total of respondents, 111(38.50%) and 218(24.30%) had symptoms of depression and anxiety respectively (Fig. 1).

### Psychosocial, behavioral, and life stressors characteristics of the study participants

According to this study finding, two-thirds (66.0%), and 98 (34%) of the prisoners had received strong social support, and poor social support respectively. The majority of the participants 169(58.7%) have stayed in prison for less than 4 years. Concerning substance use, from the participant, 135(46.9%) prisoners had substance use in their lifetime, from these substances, chewing khat 72(25%), alcohol 70(24.3%), and smoking cigarette 46(16%) were reported. From the total of respondents, 171(59.4%) had experienced at least one stressful life event in the past year (Table 2).

**Table 2:** Psychosocial, behavioral, and life stressors characteristics among prisoners in Dessie Correctional institution, Dessie, Northeast, Ethiopia, 2020 (*N* = 288)

Variable	Category	Frequency	Percent (%)
Social support	Poor social support	98	34
	Strong social support	190	66
Length of stay	< 4 years	169	58.7
	5-10 years	90	31.3
	11–24 years	29	10.1
Substance use	Ever substance use	135	46.9
	Ever khat chewing	72	25
	Ever alcohol use	70	24.3
	Ever smoking cigarette	46	16
Stressful life events	Yes	171	59.4
	No	117	40.6

### Prevalence of suicidal behavior among prisoners in Dessie correctional institution

According to our study, the prevalence of suicidal behavior was 25.3% [(95% CI: 20.5,30.6)]. Of the participants, suicidal ideation 36(12.5%), plan 22(7.6%), and 24(8.3%) had attempted in their lifetime. The most commonly used method for the suicidal attempt was hanging 9(3.1%) followed by poisoning 8(2.8%) (Table 3).

**Table 3:** Suicidal behavior among prisoners in Dessie correctional institution, Dessie, Northeast, Ethiopia, 2020 (*N* = 288)

Variable	Category	Frequency	Percentage (%)
Suicidal thought	Yes	36	12.5
	No	252	87.5
Suicidal plan	Yes	22	7.6
	No	266	92.4
Attempt(ever)	Yes	24	8.3
	No	264	91.4
Methods	Hanging	9	3.1
	Poisoning	8	2.8
	Sharp instrument	4	1.4
	Jump	1	0.3
	None	266	92.4
**The overall prevalence of suicidal behavior**	**Yes**	**73**	**25.3**
	**No**	**215**	**74.7**

### Factors associated with suicidal behavior among prisoners

In the bivariate analysis, age, sex, marital status, educational status, depression, anxiety, social support, stressful events, and ever substance use showed a *p*-value of < 0.25 and became a candidate for multivariable analysis. Sex, depression, anxiety, stressful events, and ever substance use were found to be statistically associated with suicidal behavior at a *p*-value less than 0.05.

The odds of suicidal behavior among participants with being female was 5.14 times higher as compared to males [AOR = 5.14;95% CI (1.62,16.29)]. Those prisoners with depression were nearly 5 times more likely to have suicidal behavior than their counterparts [AOR = 4.97;95% CI (2.53,9.77)]. Those prisoners who had anxiety symptoms were 3.14 times more likely to have suicidal behavior as compared with respondents who did not have anxiety symptoms [AOR = 3.14; 95% CI (1.59,6.22)]. Likewise, participants with stressful events were 5.11 times more likely to have suicidal behavior as compared with prisoners who had not experienced stressful life events [AOR = 5.11; 95% CI (2.24, 11.65)]. Finally, prisoners who used substances were 2.83 times more likely to have suicidal behavior as compared with those who did not use substances [AOR = 2.83; 95% CI (1.41, 5.59)] (Table 4).

**Table 4:** Bivariate and multivariable logistic regression analysis results of suicidal behavior among prisoners in Dessie correctional institution, Dessie, Northeast, Ethiopia, 2020 (*N* = 288)

Variables		Suicidal behavior		COR(95%C.I)	AOR(95%C.I)	*P* values
		**Yes**	**No**			
Age	18–27	30(24.6%)	92(75.4%)	0.41(0.11,1.62)	0.63(0.13,3.12)	0.57
	28–37	25(21.9%)	89(78.1%)	0.35(0.09,1.41)	0.36(0.07,1.79)	0.214
	38–47	14(32.6%)	29(67.4%)	0.60(0.14,2.60)	1.32(0.25,7.00)	0.74
	> 47	4(44.4%)	5(55.6%)	1	1	
Sex	Female	9(40.9%)	13(59.1%)	2.18(0.89,5.35)	5.14(1.62,16.29)	0.005^*^
	Male	64(24.1%)	202(75.9%)	1	1	
Marital status	Single	36(27.3%)	96(72.7%)	1.72(0.92,3.21)	2.08(0.95,4.59)	0.068
	Divorced/Separated	18(36.0%)	32(64.0%)	2.57(1.20,5.52)	1.92(0.76,4.85)	0.167
	Married	19(17.9%)	87(82.1%)	1	1	
Educational status	Unable to read & write	6(15.8%)	32(84.2%)	0.46(0.16,1.26)	0.42(0.11,1.55)	0.19
	Primary	36(30.3%)	83(69.7%)	1.06(0.55,2.04)	0.77(0.33,1.82)	0.559
	Secondary	11(17.7%)	51(82.3%)	0.53(0.23,1.216)	0.39(0.14,1.11)	0.078
	College and above	20(29.0%)	49(71.0%)	1	1	
Depression	Yes	52(46.8%)	59(53.2%)	6.54(3.64,11.80)	4.97(2.53,9.77)	< 0.0001^*^
	No	21(11.9%)	156(88.1%)	1	1	
Anxiety	Yes	36(51.4%)	37(17.0%)	5.18(2.88,9.32)	3.14(1.59,6.22)	0.001^*^
	No	34(48.6%)	181(83.0%)	1	1	
Social support	Poor	30(30.6%)	68(69.4%)	1.51(0.87,2.61)	1.22(0.60,2.47)	0.57
	Strong	43(22.6%)	147(77.4%)	1		
Stressful events	Yes	61(35.7%)	110(64.3%)	4.85(2.47,9.52)	5.11(2.24,11.65)	< 0.0001^*^
	No	12(10.3%)	105(89.7%)	1	1	
Ever use of substance	Yes	43(31.9%)	92(68.1%)	1.92(1.11,3.28)	2.83(1.41,5.69)	0.004^*^
	No	30(19.6%)	123(80.4%)	1	1	

^*^Statistically significant at *P*-value < 0.05, AOR (Adjusted odds Ratio),1 = reference category, Chi square = 8, Hosmer Lemeshow goodness-of-fit 0. 56,& degrees of freedom = 8, maximum VIF = 2.3

## Discussion

In this study, the overall prevalence of suicidal behavior was found to be 25.3%. While, the lifetime prevalence of suicidal ideation, plan, and attempts were 12.5%, 7.6%, and 8.3% respectively.

The prevalence of suicidal ideation among prisoners in this study was in line with other studies done in the USA 16% [34], Taiwan (12.5%) [35], Great Britain (14.9%) [36], and Australia (16%) [37]. However, in some other studies, such as those conducted in Chicago (53.7%) [38], Flemish Belgium (44.4%) [39], Jimma, Ethiopia 16.8% [17], Australia 34% [14], Italy 43.7% [40], China 70% [41], Iran (44.6%) [42], and Belgium 43.1% [22], the proportion of suicidal ideation was higher than in the current study. The possible reasons might be due to the difference in assessment tool in which a previous study Paykel suicidal scale (PSS) was used in Belgium [39], MINI in Italy [40], Symptoms Check List-90-Revised (SCL-90-R) in Iran [42], and Suicide Ideation scales (SSI) in China [41], whereas in this study SBQ-R was used [26]. Another possible reason for the discrepancy is that the New South Wales Australia study used stratified sampling among a sample of 996 people who completed a telephone survey [14], socio-cultural perceptions among those who expressed suicidal thoughts among 1,326 prisoners in Flemish Belgium [39], and the Chicago study used 1,418 female arrestees in awaiting trial [38]. Furthermore, these disparities could be explained by differences in socio-cultural viewpoints among those who reported suicide thoughts [43, 44]. However, the prevalence of suicide ideation is higher than in the study conducted in Addis Ababa, Ethiopia in which was found to be 8.04% [16]. The discrepancy could be due to the presence of suicidal ideation in the month leading up to the interview.

The finding of suicidal attempt in this study is in line with studies conducted in Jimma, Ethiopia (9.3%) [17], Kent State (11.9%) [37], and Italy (12.8%) [45]. However, this result was lower than other studies conducted in Belgium 20.3% [39], Australia 21% [14], Iran 38.9% [42], and Flemish Belgium (21.8%) [38], Northern Russia (17.6%) [46]. The sampling technique utilized could be the cause of the differences. For instance, in Australia stratified sampling with a telephone survey. It could be because the Russian study employed K-SADS-PL as a measuring tool [46]. The variance is also due to discrepancies in the methods employed and sociodemographic characteristics. Whereas the prevalence of suicidal attempt was higher than the study conducted in Taiwan (4.1%) [35], Australia (3.6%) [47], and Great Britain (4.4%) [47]. This disparity could be explained by the fact that the Taiwan study utilized only male inmates and used the Brief Symptom Rating Scale (BSRS-5) as a measurement tool, but great British used female but the Great Britain study used female inmates and used stratified sample with 535 inmates and used a case control study. Furthermore, this discrepancy could be explained by differences in socio-cultural viewpoints among participants who reported suicidal thoughts.

In terms of suicidal intent, the current study findings are in line with those of a previous study conducted in Jimma, Ethiopia 11.4% [17]. However, it is lower than a study done at Flemish prison reported (30.2%) [39]. The difference could be related to the fact that the Flemish study used 1326 convicts and used different tools. However, the findings of this study are higher than those of studies done among the rural general population in Butajira, which revealed that (3.2%) of the population had a suicidal plan [48]. This may be due to the obvious fact that our study was conducted in a prison in contrary to the above-mentioned community-based studies in Butajira Ethiopia, because being imprisoned is itself a stressful event for even healthy inmates, and are at increased risk for suicide since it deprives the person of important resources [24]. This could be due to the fact that, in contrast to the above-mentioned community-based studies in Butajira Ethiopia, our study was conducted in a prison, because being incarcerated is a stressful event for even healthy inmates, who are at an increased risk of suicide because it deprives them of important resources.

Regarding the associated factors, in this study, being females were 5.14 times more likely to have suicidal behavior as compared to males. This finding was supported by research conducted in Israel [49], and Ethiopia [50]. This could be due to differences in the biological makeup, socio-cultural influences on women's ability to express their issues compared to men, and suppressed emotion that can lead to suicide tendencies [16].

We found that participants who had depression were 5 times more likely to have suicidal behavior than undepressed participants. Similar to a finding of different studies from New South Wales Australia [14], Taiwan [35], British [51], and Jimma, Ethiopia [17]. This could be because depressed people have lower levels of the neurotransmitter serotonin in their brain, which can lead to feelings of hopelessness worthlessness, and guilt, which can lead to suicidal behavior [7]. It could also be because jail adds to their stress, negatively impacting their mental health and possibly increasing existing psychopathology [24].

This finding also revealed that prisoners with anxiety were 3.14 times more likely to have suicidal behavior than their counterparts. This was supported by the study conducted in Flemish Belgium [39], and Chicago [38]. Fear of adjusting to a new environment, as well as an increase in psychosocial stress, can lead to anxiety, which, in turn, can lead to suicide behavior [52].

Another predictor for suicidal behavior was the stressful events, those prisoners who had experienced stressful events were 5.11 times more likely to have suicidal behavior as compared with participants who do not experience stressful events. A current study finding was congruent with another finding [53]. This could be because, as many studies have shown, incarcerated people face a wide range of stressful situations throughout their lives, including physical or sexual abuse, unsuccessful court decisions, the separation from children, the end of partner relationships, financial difficulties, and life-threatening accidents, all of which can lead to stressful situations that force them to engage in suicidal behavior [54].

Finally, in this study, prisoners who use substance were 2.83 times more likely to have suicidal behavior as compared with those who did not use substance. This finding is in line with other the study [14]. This study result revealed that substance use is one risk factor for suicide. Substance users have repeated episodes of behavioral disturbance due to the effects of acute intoxication and self-medication and poor social relationships which cause suicidal behavior [55].

## Limitations of the study

First, the data was collected based on retrospective self-report and thus may be subject to social desirability and recall bias. Secondly, due to its cross-sectional nature, the study could not explore the cause and effect relationship of variables. Finally, the participants of the study were recruited from only in Dessie correctional institution, restricted to presenting an enormous amount of information on the prevalence rate of suicidal behavior among the different populations of the Ethiopian prison.

## Conclusions

In this study, we examined the prevalence and important predictors of suicidal behavior among incarcerated in Dessie town correctional center. The result noted that nearly one-fourth of prisoners reported suicidal behavior (suicidal ideation, plan, and attempt). This study indicates that it is an important public health problem. These factors include being female, depression, anxiety, stressful life events, and substance use were identified as independent predictors for suicidal behavior among incarcerated in prison. Despite the fact that, our study did not allow us to establish a temporal relationship supported by strong scientific evidence, we concluded that prison health professionals should pay close particular attention to early screening and treatment of suicidal behavior among people who are incarcerated.

## Data Availability

All raw data included in the current study can be accessed from the corresponding author through the email address of a rational request. The data sets of the current study are available from [Tamrat Anbesaw], Email: tamratanbesaw@gmail.com; Mobile: + 251(0)9–11,289,143, Wollo University, Dessie upon reasonable request.

## Abbreviations

CI: Confidence Interval
HADS: Hospital Anxiety Depression Scale
KM: Kilometers
LAMIC: Low and middle-income countries
MINI: Mini-International Neuropsychiatric Interview
OR: Odds Ratio
PSS: Paykel suicidal scale
SBQ-R: Suicidal Behavior Questionnaire-Revised
SD: Standard Deviation
SPSS: Statistical Package for the Social Sciences
UK: United Kingdom
US: United State
USA: United States of America
WHO: World Health Organization
